# Long-Term Mixed Chimerism After *Ex Vivo*/*In Vivo* T Cell-Depleted Allogeneic Hematopoietic Cell Transplantation in Patients With Myeloid Neoplasms

**DOI:** 10.3389/fonc.2021.776946

**Published:** 2021-12-07

**Authors:** Leo Ruhnke, Friedrich Stölzel, Uta Oelschlägel, Malte von Bonin, Katja Sockel, Jan Moritz Middeke, Christoph Röllig, Korinna Jöhrens, Johannes Schetelig, Christian Thiede, Martin Bornhäuser

**Affiliations:** ^1^ Department of Internal Medicine I, University Hospital Dresden, TU Dresden, Dresden, Germany; ^2^ German Cancer Consortium (DKTK) partner site Dresden, Dresden, Germany; ^3^ German Cancer Research Center (DKFZ), Heidelberg, Germany; ^4^ Institute of Pathology, University Hospital Dresden, TU Dresden, Dresden, Germany; ^5^ DKMS Clinical Trials Unit, Dresden, Germany; ^6^ AgenDix GmbH, Dresden, Germany; ^7^ National Center for Tumor Diseases (NCT) Dresden, Dresden, Germany

**Keywords:** chimerism, mixed donor chimerism, allogeneic hematopoietic cell transplantation, myeloid neoplasms, AML, CML

## Abstract

In patients who have undergone allogeneic hematopoietic cell transplantation (HCT), myeloid mixed donor chimerism (MC) is a risk factor for disease relapse. In contrast, several studies found favorable outcome in patients with lymphoid MC. Thus far, most studies evaluating MC focused on a *short-term* follow-up period. Here, we report the first case series of *long-term* survivors with MC. We screened 1,346 patients having undergone HCT for myeloid neoplasms at our center from 1996 to 2016; 443 patients with data on total peripheral blood mononuclear cells (PBMC)/CD4^+^/CD34^+^ short tandem repeat (STR) donor chimerism (DC) and follow-up ≥24 months post-HCT were included. We identified 10 patients with *long-term* MC (PBMC DC <95% at ≥12 months post-HCT). Median follow-up was 11 years. All patients had received combined *ex vivo/in vivo* T cell-depleted (TCD) peripheral blood stem cells; none experienced ≥grade 2 acute graft-versus-host disease (GVHD). The mean total PBMC, CD4^+^, and CD34^+^ DC of all patients were 95.88%, 85.84%, and 90.15%, respectively. Reduced-intensity conditioning (RIC) was associated with a trend to lower mean total DC. Of note, two patients who experienced relapse had lower CD34^+^ DC but higher CD4^+^ DC as compared with patients in continuous remission. Bone marrow evaluation revealed increased CD4^+^/FOXP3^+^ cells in patients with MC, which might indicate expansion of regulatory T cells (T_regs_). Our results support known predictive factors associated with MC such as RIC and TCD, promote the value of CD34^+^ MC as a potential predictor of relapse, highlight the potential association of CD4^+^ MC with reduced risk of GVHD, and indicate a possible role of T_regs_ in the maintenance of immune tolerance post-HCT.

## Introduction

The rationale of allogeneic hematopoietic cell transplantation (HCT) for hematologic malignancies is complete eradication of malignant clones and subsequent replacement of the recipients by the donor hematopoiesis together with development of full donor chimerism (FDC). However, some patients—transplanted for either malignant or non-malignant hematologic disorders—retain recipient hematopoietic cells and develop a mixture of both donor- and recipient-derived hematopoiesis, referred to as mixed donor chimerism (MC). Several factors for the establishment of MC have been identified, e.g., the underlying disease [inherited bone marrow failure syndromes (IBMFS) vs. hematologic malignancies, myeloid vs. lymphoid neoplasms)]; conditioning regimen [reduced-intensity conditioning (RIC) vs. myeloablative conditioning (MAC)]; use of T-cell depletion (TCD) (*ex vivo*, e.g., CD34^+^ selection; or *in vivo*, e.g., anti-thymocyte globulin (ATG) or alemtuzumab); and the dose of CD34^+^ cells infused (1–6). In patients with IBMFS, such as thalassemia, presence of MC is a common event after successful transplantation. Here, the outcome of patients transplanted for IBMFS with persistent MC seems to be comparable with that with FDC (7, 8). In contrast, in hematologic malignancies, MC is a risk factor for graft failure and relapse (9). For example, in a study conducted by Lamba and colleagues, patients with day +90 peripheral blood mononuclear cells (PBMC) MC had significantly higher relapse rates as compared with patients with FDC at 6 months post-HCT (23% vs. 7%; *p* = .03) (10). Similar, Koreth et al. found that patients with day +100 PBMC MC had higher 2-year relapse rates (53% vs. 30%; *p* <.0001) and worse 2-year progression-free survival (PFS) (37% vs. 59%; *p* <.0001) as compared with patients with day +100 PBMC FDC (11). In particular, a decline in DC/MC of the myeloid lineage (CD33^+^/CD34^+^) is associated with disease relapse: in patients transplanted for acute myeloid leukemia (AML) and myelodysplastic syndrome (MDS), CD34^+^ PBMC MC was found to be an independent predictor of relapse and inferior survival (12–14). Here, the incidence of relapse in patients with CD34^+^ MC was 57% as compared with 18% in patients with CD34^+^ FDC (12). The impact of lymphoid MC is still under discussion. While Koreth et al. found CD3^+^ MC to be associated with increased rates of relapse and unfavorable PFS and overall survival (OS) in patients with hematological malignancies undergoing T cell-replete HCT, Deeg and colleagues reported improved PFS and OS in patients with CD3^+^ MC who underwent HCT for primary or secondary myelofibrosis (11, 15). However, there is evidence that low-grade MC—in particular MC affecting the lymphoid lineage (CD3^+^/CD4^+^)—is associated with lower non-relapse mortality (NRM): in line with previous reports, Baron et al. showed that lymphoid MC is associated with a lower rate of grade 2–4 acute graft-versus-host disease (GVHD) in patients who underwent RIC (9, 16, 17). Here, Sykes and colleagues evaluated the intentional induction of MC aiming for the induction of immune tolerance in patients undergoing HCT: their novel RIC/TCD (ATG/thymic irradiation) regimen was associated with a low rate of NRM and grade 2–4 acute GVHD and enabled safe subsequent administration of donor lymphocytes (18, 19). Of note, most studies evaluating chimerism focus on a *short-term* follow-up period (≤12–24 months post-HCT). Accordingly, little is known about patients with *long-term* MC. Here, we aimed to evaluate incidence, risk factors, and associated patient outcome of *long-term* MC in patients who underwent HCT for myeloid neoplasms.

## Methods

For this retrospective analysis, all patients who underwent allogeneic HCT for myeloid neoplasms at our center from 1996 to 2016 (n = 1,346) were screened for *long-term* MC. **Supplementary Figure 1** summarizes the patient selection and composition. Four hundred forty-three patients with myeloid neoplasms with follow-up ≥24 months post-HCT and data on short tandem repeat (STR)-based PBMC, lymphoid (CD4^+^), and myeloid (CD34^+^) subset DC at various timepoints post-HCT were included in the present analysis. Patients with evidence of molecular (other than MC) or hematological relapse within 12 months post-HCT were excluded. *Long-term* MC was defined as PBMC DC <95% at ≥12 months post-HCT. All patients provided written informed consent; forms were approved by the Ethics Committee of the TU Dresden (EK98032010) in accordance with the Declaration of Helsinki.

STR-based DC analysis had been performed on PBMC and/or bone marrow mononuclear cells (BMMC) on a regular basis according to institutional standards (PBMC-DC: every 14 days for the first 4 months, monthly for eight additional months, and then every 3 months and as indicated/needed (e.g., new blood cytopenia); BMMC-DC: around day +90 and day +180 post-HCT and as indicated/needed (e.g., new blood cytopenia)). DC was determined using the AmpFlSTR Profiler PCR Amplification Kit (Applied Biosystems, Darmstadt, Germany) (20). From 2004 on, the Mentype^®^ Chimera^®^ Kit (Biotype, Dresden, Germany), which uses STR loci (D2S1360, D3S1744, D4S2366, D5S2500, D6S474, D7S1517, D8S1132, D10S2325, D12S391, D18S51, D21S2055, and SE33 (ACTBP2)) and the amelogenin locus (on the X and Y chromosomes), was used as previously reported (21).

Patients with relapse, measurable residual disease (MRD)-positive patients, and/or patients with MC were evaluated for donor lymphocyte infusion (DLI) on a regular basis according to institutional standards.

Immunohistochemistry (IHC) in formalin-fixed paraffin-embedded (FFPE) bone marrow trephine biopsies were performed as previously described (22). Bone marrow biopsies obtained >12 months post-HCT were available for three patients (UPN01, UPN04, and UPN06), bone marrow biopsies from three patients in continuous complete remission (CR) and with FDC >12 months post-HCT served as control. The following primary antibodies were used: CD4 (clone SP35, Ventana/Roche Diagnostics), FOXP3 (clone SP97, Abcam), PDCD1 [PD1] (clone NAT105, Ventana/Roche Diagnostics), CD274 [PD-L1] (clone SP263, Ventana/Roche Diagnostics), and HAVCR2 [TIM3] (clone EPR22241, Abcam).

Standard statistical methods were used for descriptive analyses, the Mann–Whitney *U* test was used for non-parametric data, and the Shapiro–Wilk test was used to test for normal distribution; analyses were done with R version 3.5.3, RStudio version 1.2.1335 and GraphPad Prism version 9.0.0.

## Results

**Figure 1:**
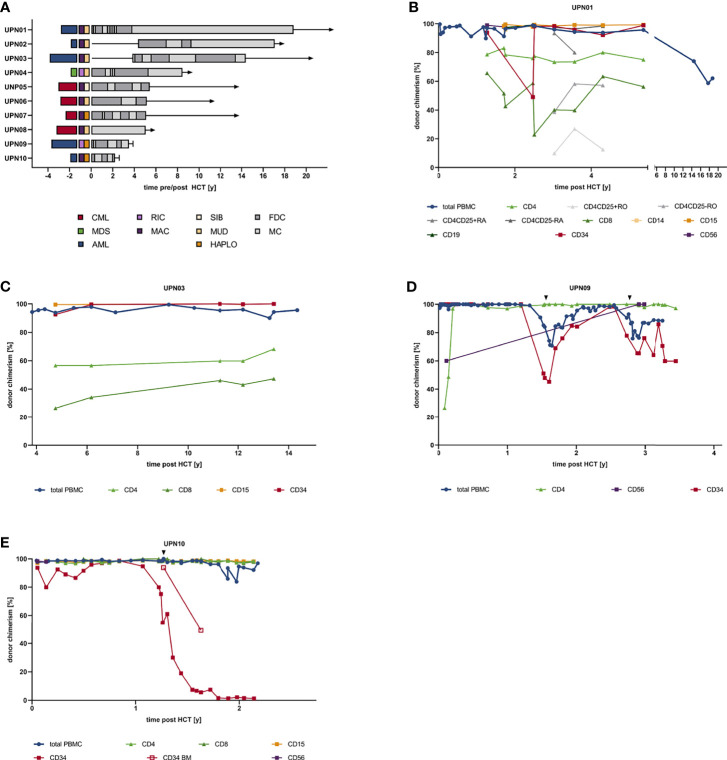
Patient and transplant characteristics and individual donor chimerism kinetics. **(A)** Swimmer plot indicating diagnosis, transplant characteristics, and chimerism kinetics. **(B)** Chimerism kinetics of UPN01. **(C)** Chimerism kinetics of UPN03. **(D)** Chimerism kinetics of UPN09. **(E)** Chimerism kinetics of UPN10. AML, acute myeloid leukemia; CML, chronic myeloid leukemia; FDC, full donor chimerism; HAPLO, haploidentical donor; HCT, hematopoietic cell transplantation; MAC, myeloablative conditioning; MC, mixed chimerism; MDS, myelodysplastic syndrome; MUD, matched unrelated donor; RIC, reduced-intensity conditioning; SIB, sibling donor; UPN, unique patient number.

We identified 10 patients [0.74% of all patients who have undergone HCT for myeloid neoplasms (n = 1,346) and 2.26% of patients with valid records and follow-up ≥24 months (n = 443)] with *long-term* MC (<95% DC at ≥12 months post-HCT). Patient demographics and transplantation characteristics are summarized in **Table 1** and **Figure 1A**. Of these patients, one was diagnosed with MDS, five patients (50%) were diagnosed with AML, and four patients (40%) were diagnosed with chronic myeloid leukemia (CML). The time from diagnosis to allogeneic HCT ranged from 4 to 27 months (median, 11 months). The median age at transplantation was 39 years (range, 19–66 years). The median follow-up was 11 years (range, 2.2–21 years). Six patients (60%) had a human leukocyte antigen (HLA)-matched unrelated donor (MUD), three patients (30%) had an HLA-haploidentical donor (HAPLO), and one patient had an HLA-identical sibling who served as donor (SIB). All patients were cytomegalovirus (CMV) seronegative, and only one patient received a CMV-seropositive graft. The conditioning regimen was RIC/non-myeloablative in two patients (20%). Myeloablative conditioning was used in eight patients (80%); amongst these, five patients (45%) received fractionated total body irradiation (TBI) to a total dose of 12 Gy. Of note, all patients received combined *ex vivo* (CD34-selection or CD3/CD19-depletion)/*in vivo* (ATG, alemtuzumab, muromonab-CD3) TCD peripheral blood stem cells (PBSC). The median CD3^+^ and CD34^+^ cell dose was 3.11 × 10^3^/kg and 6.54 × 10^6^/kg, respectively. For GVHD prophylaxis, all patients received *in vivo* TCD (ATG, alemtuzumab, or muromonab-CD3) and calcineurin inhibitors with or without mycophenolate mofetil. All patients transplanted for CML received DLIs based on the institutional standards. None of the patients experienced ≥grade 2 acute GVHD or moderate/extensive chronic GVHD. At the last follow-up, eight patients (80%) were alive in continuous complete remission (CR), while two patients (UPN 09, UPN 10) deceased due to relapse of the underlying malignancy 26 and 45 months post-HCT.

**Table 1:** Patient and transplant characteristics.

UPN	Year of HCT	Disease	Age at HCT	Gender(D/R)	CMV(D/R)	Donor	Stem cell source	Conditioning regimen	Conditioning protocol	*In vivo* TCD	Graft manipulation	GVHD prophylaxis	DLI/NK	aGVHD grade^†^	cGVHD severity^‡^	Outcome at last follow-up
01	1998	AML	27	M/M	N/N	MUD	PBSC	MAC	Bu/Cy/Eto	ATG	CD34 selection	CNI	–	1	Mild	CR, ECOG 0
02	1997	AML	29	F/M	N/N	MUD	PBSC	MAC	Bu/Cy	ATG	CD34 selection	CNI	–	0	–	CR, ECOG 0
03	1997	AML	35	M/M	N/N	MUD	PBSC	MAC	Bu/Cy	ATG	CD34 selection	CNI	–	0	–	CR, ECOG 0
04	2008	MDS	19	M/M	N/N	MUD	PBSC	RIC	2 Gy TBI/Cy	ATG	CD34 selection	CNI/MMF	–	0	–	CR, ECOG 0
05	2005	CML	36	M/F	N/N	SIB	PBSC	MAC	12 Gy TBI/Flu/Thio	ATG	CD34 selection	CNI*	DLI^#^	1	–	CR, ECOG 0
06	2006	CML	66	M/M	N/N	MUD	PBSC	MAC	8 Gy TBI/Flu/Thio	ATG	CD34 selection	CNI*	DLI^#^	1	Mild	CR, ECOG 0
07	2001	CML	32	F/M	P/N	HAPLO	PBSC	MAC	12 Gy TBI/Flu/Thio	ATG	CD34 selection	CNI*	NK	0	–	CR, ECOG 0
08	2006	CML	50	M/M	N/N	MUD	PBSC	MAC	12 Gy TBI/Flu	ATG	CD34 selection	CNI*	DLI^#^	1	Mild	CR, ECOG 0
09	2008	AML	57	M/M	N/N	HAPLO	PBSC	RIC	Mel/Flu/Thio	Muromonab-CD3	CD34 selection	–	–	0	0	Deceased due to relapse
10	2002	AML	43	M/F	N/N	HAPLO	PBSC	MAC	12 Gy TBI/Flu/Thio	ATG	CD34 selection	CNI*	–	0	0	Deceased due to relapse

AML, acute myeloid leukemia; ATG, anti-thymocyte globulin; Bu, busulfan; CML, chronic myeloid leukemia; CNI, calcineurin inhibitor; CR, complete remission; Cy, cyclophosphamide; DC, donor chimerism; DLI, donor lymphocyte infusion; D/R, donor/recipient; Eto, etoposide; F, female, Flu, fludarabine; GVHD, graft-versus-host disease; HAPLO, haploidentical donor; HCT, hematopoietic cell transplantation; M, male, MAC, myeloablative conditioning; MDS, myelodysplastic syndrome; Mel, melphalan; MMF, mycophenolate mofetil; MUD, matched unrelated donor; NK, natural killer cell administration; PBSC, peripheral blood stem cells; RIC, reduced-intensity conditioning; SIB, sibling donor; TBI, total body irradiation; TCD, T-cell depletion; Thio, thiotepa; UPN, unique patient number; ECOG, Eastern Cooperative Oncology Group.

^†^Maximum aGVHD grade according to (23).

^‡^Maximum cGVHD grade according to (24).

^*^Throughout conditioning; ^#^d56/112.

**Figure 2:**
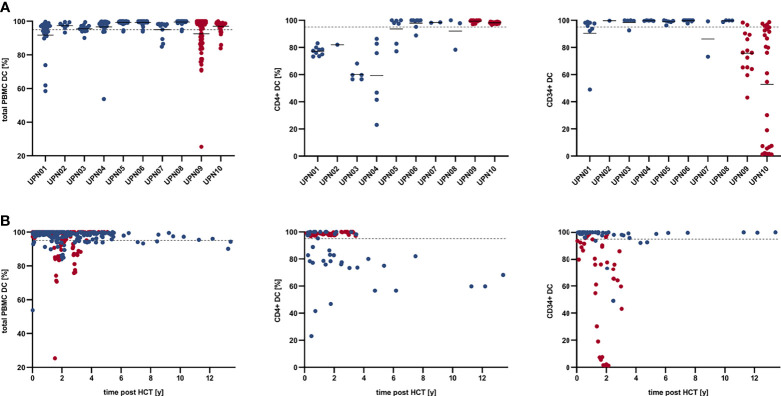
Total PBMC, CD4^+^, and CD34^+^ donor chimerism kinetics in MC patients who are alive in continuous CR (blue) and MC patients who deceased due to relapse (red). **(A)** Total PBMC, CD4^+^, and CD34^+^ donor chimerism per patient; solid lines indicate mean total PBMC, CD4^+^, and CD34^+^ DC per UPN. **(B)** Total PBMC, CD4^+^, and CD34^+^ donor chimerism over time post HCT. Dashed line indicates 95% DC. CR, complete remission; DC, donor chimerism; HCT, hematopoietic cell transplantation; MC, mixed chimerism; PBMC, peripheral blood mononuclear cells; UPN, unique patient number.

Patients with >10% mixed CD4/CD34^+^ DC (UPN01, UPN03, UPN09, and UPN10) and the management of these patients were assessed in detail. In UPN01, the diagnosis of acute promyelocytic leukemia [APL with t (15, 17) (q22;q21); *PML*–*RARA* fusion (bcr3)] was established. Intensive induction and consolidation therapy were performed according to the APL93 trial protocol (NCT00599937). Two years after achieving a CR, the patient experienced relapse, and re-induction therapy and subsequent allogeneic HCT with an *ex vivo/in vivo* TCD graft (CD34 selection + ATG) from a matched unrelated donor was performed. Follow-up examinations showed complete hematological and molecular remission for the last 20 years but repeatedly revealed MC affecting the lymphoid as well as myeloid lineage (**Figures 1B**, **2**). UPN03 (**Figures 1C**, **2**) was diagnosed with therapy-related AML (prior radiotherapy for testicular germ cell tumor; normal male karyotype) in 1994. After induction therapy, the patient achieved CR and received consolidation and maintenance therapy. However, shortly thereafter, the patient experienced relapse, received re-induction therapy, and underwent subsequent allogeneic HCT with an *ex vivo/in vivo* TCD graft (CD34 selection + ATG) from a MUD in CR2. Notwithstanding, with regard MC, especially affecting the lymphoid lineage, the patient is still in complete hematological remission. UPN09 (**Figures 1D**, **2**) was diagnosed with secondary AML (prior diagnosis of myeloproliferative neoplasm (MPN); AML not otherwise specified (NOS), AML without maturation, normal male karyotype, no *BCR*–*ABL1* fusion, no *CEBPA*, *FLT3*, or *NPM1* mutation) in 2008. The patient received intensive induction therapy and underwent haploidentical HCT with an *ex vivo/in vivo* TCD graft (CD3/CD19 depletion + muromonab-CD3) in CR1. Eighteen months later, the patient relapsed and received a total of 23 courses of azacytidine over 2 years, which resulted in a CR with incomplete hematologic recovery (CRi). In 2012, he relapsed again and received intensive re-induction chemotherapy and underwent second haploidentical HCT, which was complicated by pneumonia with fatal outcome during aplasia post-HCT. Both relapses were accompanied by prior decline in CD34^+^ DC, while full CD4^+^ DC was observed. UPN10 (**Figures 1E**, **2**) was diagnosed with AML (AML with inv (3)(q21.3q26.2); *GATA2*, *MECOM*; ELN adverse risk category) in 2002 and received intensive induction therapy and subsequently underwent allogeneic haploidentical HCT with an *ex vivo/in vivo* TCD graft (CD34 selection + ATG) in CR1 3 months later. Fifteen months later, the patient relapsed; under salvage chemotherapy, morphologic leukemia-free state (MLFS) was achieved. However, the patient died due to recurring infectious complications under best supportive care measures 27 months post-HCT. Again, relapse was associated with CD34^+^ PBMC/BMMC MC, while in CD4/CD8^+^ subsets, FDC could be observed.

**Figure 3:**
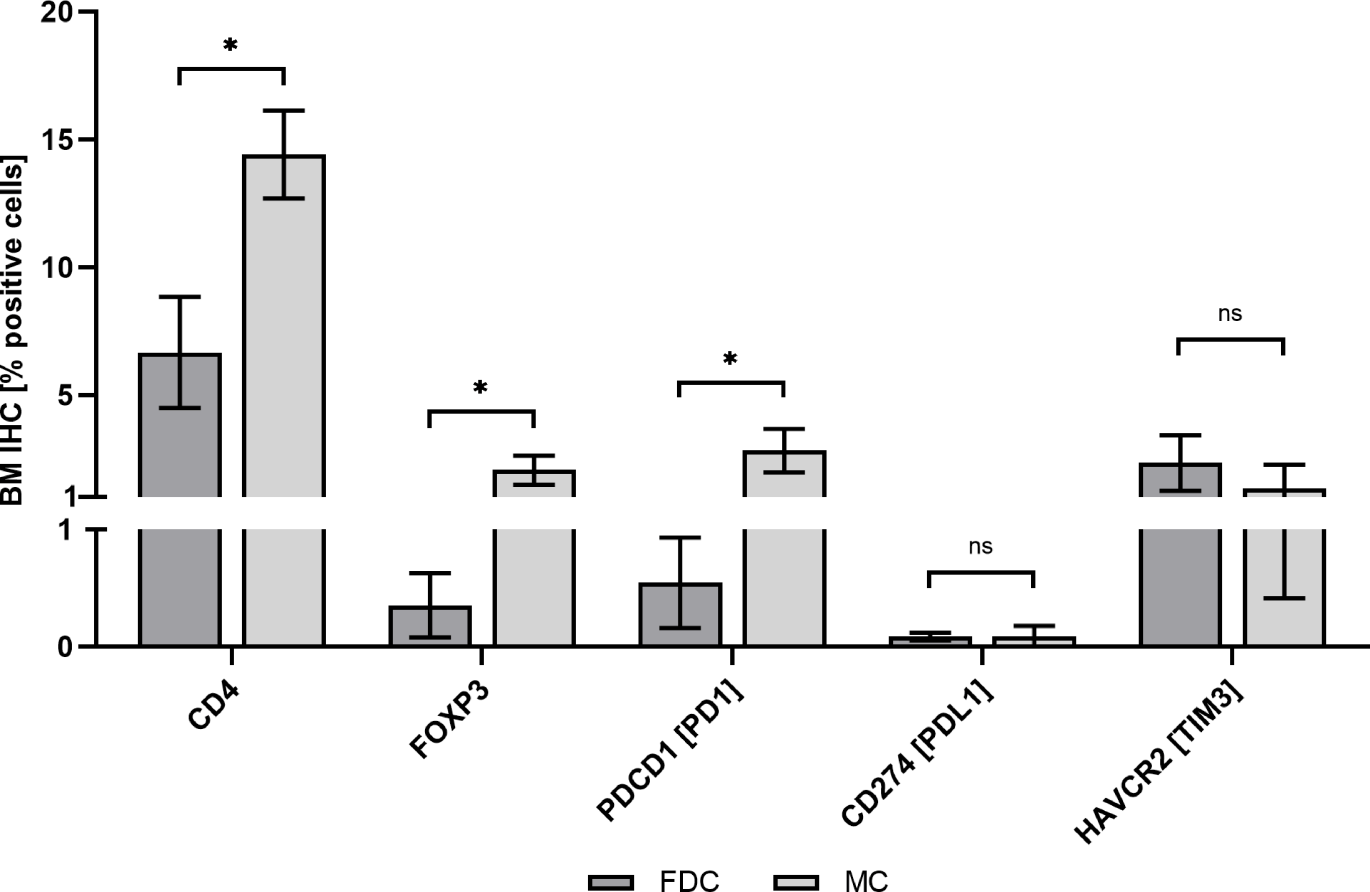
Bone marrow immunohistochemistry assessment. Patients with FDC (n = 8) vs. patients with MC (n = 3). BM, bone marrow; FDC, full donor chimerism; IHC, immunohistochemistry; MC, mixed chimerism; ns, non-significant; *p < .05.

Overall, patients with CD4^+^ MC and CD34^+^ FDC (n = 6), CD4^+^ FDC and CD34^+^ MC (n = 2), and CD4^+^ and CD34^+^ MC (n = 2) were identified. **Table 2** and **Figure 2** summarize the results of individual chimerism determinations for total DC, CD4^+^ DC, and CD34^+^ DC. The mean total PBMC DC, CD4^+^ DC, and CD34^+^ DC of all patients was 95.88% (range, 90.56%–99.52%), 85.84% (range, 59.40%–99.38%), and 90.15% (range, 52.84%–99.76%), respectively. RIC was associated with a trend to lower mean total PBMC DC as compared with MAC (91.57% vs. 98.71%, *p* = .0714). Patients with haploidentical donors demonstrated lower mean CD34^+^ DC (71.63% vs. 98.08% *p* = .0176) but higher mean CD4^+^ DC (98.63% vs. 80.36%, *p* = .0176). Of note, the two patients who experienced relapse (UPN09 and UPN10) had lower mean CD34^+^ DC (64.32% vs. 96.61%, *p* = .0444) but higher CD4^+^ DC (98.78% vs. 82.61%, *p* = .0444) as compared with patients in continuous CR. Patient age, patient gender, myeloid neoplasm (AML vs. CML), time from initial diagnosis to allogeneic HCT, and CD3^+^/CD34^+^ cell dose had no significant impact on the extent of MC in either CD4^+^ or CD34^+^ cells. To address the underlying mechanisms of MC without evidence of relapse, we performed IHC studies in bone marrow trephine biopsies obtained >12 months post-HCT, which were available for three patients (UPN01, UPN04, and UPN06); eight patients in CR with FDC > 12 months post-HCT served as control. IHC showed more CD4^+^ (14.41% vs. 6.66%, *p* = .0391), FOXP3^+^ (2.06% vs. 0.36%, *p* = .0474) and PDCD1 [PD1]^+^ (2.82% vs. 0.54%, *p* =.0292) cells in patients with MC as compared with patients with FDC; in contrast, no differences in CD274 [PDL1]^+^ (0.08% vs. 0.09%, *p* = .9792) or HAVCR2 [TIM3]^+^ (1.35% vs. 2.34%, *p* = .2301) cells were observed (**Figure 3**).

**Table 2:** Donor chimerism in association with patient and transplant characteristics.

	Total DCmean (range), %	*p*-Value	CD4^+^ DCmean (range), %	*p*-Value	CD34^+^ DCmean (range), %	*p*-Value
**All patients**	95.88 (90.56–99.52)		85.84 (59.40–99.38)		90.15 (52.84–99.76)	
**Sex**						
Female	96.37 (92.58–99.30)		92.80 (77.29–99.38)		72.85 (52.84–99.71)	
Male	95.45 (90.56–99.52)	.8927	84.44 (59.40–97.95)	.4509	83.99 (75.79–99.76)	.4723
**Age**						
<50	96.38 (90.56–99.30)		91.01 (60.20–99.38)		81.56 (52.84–99.71)	
≥50	96.95 (92.58–99.52)	.8323	89.86 (59.40–98.35)	.7654	83.59 (75.79–99.76)	.6775
**Disease**						
AML	94.46 (90.56–97.42)		79.42 (60.20–98.17)		85.36 (52.84–99.70)	
CML	97.14 (92.58–99.52)	.2222	96.28 (92.10–99.38)	.1111	92.06 (75.79–99.71)	.4970
**Conditioning** **regimen**						
RIC	91.57 (90.56–92.58)		90.10 (59.40–93.60)		85.09 (75.79–99.76)	
MAC	98.71 (95.01–99.52)	.0714	91.85 (60.20–99.38)	.7267	81.44 (52.84–99.71)	.2222
**Donor**						
HAPLO	94.88 (92.58–97.04)		98.63 (98.17–99.38)		71.63 (52.84–86.25)	
Non-HAPLO	96.25 (90.56–99.52)	.4970	80.36 (59.40–97.95)	**.0167**	98.08 (90.49–99.76)	**.0176**
**CD34^+^ cell dose**						
<5 × 10^6^/kg	95.66 (90.56–99.31)		91.73 (77.29–98.35)		79.08 (52.84–99.71)	
≥5 × 10^6^/kg	96.86 (91.74–99.52)	.6202	89.33 (59.40–99.38)	.8543	81.09 (75.79.99.76)	.6754
**Relapse**						
Yes	94.81 (92.58–97.04)		98.78 (98.17–99.38)		64.32 (52.84–75.79)	
No	96.11 (90.56–99.52)	.7273	82.61 (59.40–98.07)	**.0444**	96.61 (86.25–99.76)	**.0444**

AML, acute myeloid leukemia; CML, chronic myeloid leukemia; DC, donor chimerism; HAPLO, haploidentical donor; MAC, myeloablative conditioning; RIC, reduced-intensity conditioning. Bold values indicate statistically significant p values.

## Discussion

The rationale of HCT for hematologic malignancies is the complete eradication of malignant clones and subsequent replacement of the recipients by the donor hematopoiesis, achieving FDC. Several studies found favorable outcome in patients with low-grade MC—in particular lymphoid (CD3^+^/CD4^+^) MC after allogeneic HCT (9, 16, 17). In contrast, myeloid (CD33^+^/CD34^+^) MC is associated with disease relapse and poor prognosis (12–14). Of note, most studies evaluating chimerism focus on a *short-term* follow-up period (≤12–24 months post-HCT); therefore, little is known about patients with *long-term* MC. Here, we aimed to evaluate incidence, risk factors, and associated patient outcome of *long-term* MC in patients who underwent HCT for myeloid neoplasms. While screening 1,346 patients who underwent HCT for myeloid neoplasms at our center, 10 patients with *long-term* MC (0.74% of all patients undergoing HCT for myeloid neoplasms; 2.26% of patients with valid records and follow-up ≥24 months post-HCT) were identified. In contrast to our cohort, evaluating 231 patients transplanted for hematologic malignancies and severe aplastic anemia (SAA), Schaap et al. identified 19 patients (8.26%) with long-term MC. However, most patients evaluated by Schaap and coworkers were diagnosed with acute lymphoblastic leukemia (ALL), donors were HLA-A, HLA-B, HLA-DRB1, and HLA-DQB1-identical siblings, and all patients who were evaluated received TCD grafts (2). In contrast, we focused on patients with myeloid neoplasms, and all but one patient with evidence of MC had matched-unrelated or haploidentical donors, which might explain different incidences of MC. In accordance with Schaap et al. and others, all patients with MC received *ex vivo* as well as *in vivo* TCD (2, 25, 26). For example, Grimaldi et al. reported prolonged CD4^+^ MC in patients transplanted for SAA after TCD with alemtuzumab; furthermore, several other authors found TCD with ATG or alemtuzumab to be associated with MC (2, 26–28). In accordance with previous reports, TCD and subsequent CD4^+^ MC were associated with a low incidence of GVHD: none of our patients experienced ≥grade 2 acute GVHD or extensive chronic GVHD (2, 9, 26). Furthermore, conditioning regimen had an impact on DC: patients with RIC were more likely to show mixed total PBMC DC than patients with MAC; in contrast to previous reports, RIC was associated with mixed total PBMC DC but not mixed CD4^+^/CD34^+^ DC; however, a small number of cases caution against overinterpretation of these data (9, 29). In our analysis, patients with haploidentical donors had lower total and CD34^+^, but higher CD4^+^ DC. In contrast, Federmann et al. reported achievement of FDC in all patients after haploidentical HCT with RIC and *ex vivo* TCD (30). Both patients who deceased due to relapse showed recurrent/persistent decline in CD34^+^ DC, which is in line with previous studies identifying CD34^+^ MC as an independent risk factor for subsequent relapse and inferior survival (12, 14). Here, our findings support CD34^+^ DC monitoring, in particular since preemptive intervention might prevent/delay hematologic relapse in patients after HCT (13). Patient age, patient gender, and CD3^+^/CD34^+^ cell dose had no impact on the extent of MC. To address possible mechanisms of immune tolerance in MC long-term survivors, we performed IHC in BM trephine biopsies. As compared with FDC patients, IHC showed increased CD4^+^ and FOXP3^+^ cells in patients with MC, which might indicate expansion of regulatory T cells (T_regs_) (31, 32). Here, our results are in line with previous reports: Kinsella et al. found increased levels of T_regs_ in MC patients as compared with patients with DC evaluating 69 patients with AML undergoing reduced-intensity conditioned allogeneic HCT with *ex vivo* TCD grafts (33). Of note, several studies indicate that ATG might induce T_reg_ expansion and that T_reg_ might preserve graft-versus-leukemia (GVL) effect while inhibiting GVHD (34–37). Similarly, in patients who have undergone combined kidney and bone marrow transplantation (CKBMT), donor-specific T_regs_ expanded within 6 months after CKBMT in those patients who achieved allograft tolerance, suggesting that early expansion of donor-specific T_regs_ is involved in tolerance induction following CKBMT (38). Here, we report one of the first case series of long-term survivors with MC with a follow-up >10 years post HCT. Our results support RIC and TCD as risk factors for MC and highlight the value of CD34^+^ PBMC MC as a potential independent predictor of relapse and inferior survival. While CD4^+^ MC seem to be associated with reduced risk of GVHD, the role of T_regs_ in immune tolerance induction needs to be appreciated. Since little is known about the mechanisms of MC, further research addressing mechanisms maintaining GVL while suppressing GVHD as well as the origin and function of maintained autologous non-malignant lymphohematopoietic cells is warranted.

## Data Availability Statement

The original contributions presented in the study are included in the article/**Supplementary Material**. Further inquiries can be directed to the corresponding author.

## Ethics Statement

The studies involving human participants were reviewed and approved by the Ethics Committee of the TU Dresden (EK98032010). The patients/participants provided their written informed consent to participate in this study.

## Author Contributions

LR, FS, MB, and CT conceived the presented idea. FS, MvB, CR, KS, JM, and CR took care of the patients. LR, FS, UO, MvB, KJ, and CT performed the research. LR, FS, JS, and CT performed the data analysis. LR wrote the manuscript with help from FS, UO, CR, JS, MB, and CT. All authors contributed to the article and approved the submitted version.

## Conflict of Interest

Author CT was employed by AgenDix GmbH.

The remaining authors declare that the research was conducted in the absence of any commercial or financial relationships that could be construed as a potential conflict of interest.

## Publisher’s Note

All claims expressed in this article are solely those of the authors and do not necessarily represent those of their affiliated organizations, or those of the publisher, the editors and the reviewers. Any product that may be evaluated in this article, or claim that may be made by its manufacturer, is not guaranteed or endorsed by the publisher.

## Supplementary Material

Supplementary Figure 1 Patient selection and composition. AML, acute myeloid leukemia; CML, chronic myeloid leukemia; FU, follow-up; HCT, hematopoietic cell transplantation; MDS, myelodysplastic syndrome; MPN, myeloproliferative neoplasms; PBMC, peripheral blood mononuclear cells; STR, short tandem repeats.
