# Supplementary figures and images for: Long-Term Mixed Chimerism After Ex Vivo/In Vivo T Cell-Depleted Allogeneic Hematopoietic Cell Transplantation in Patients With Myeloid Neoplasms
Source: Front Oncol. 2021 Dec 7;11:776946. doi: 10.3389/fonc.2021.776946 (PMC8688843; doi:10.3389/fonc.2021.776946)

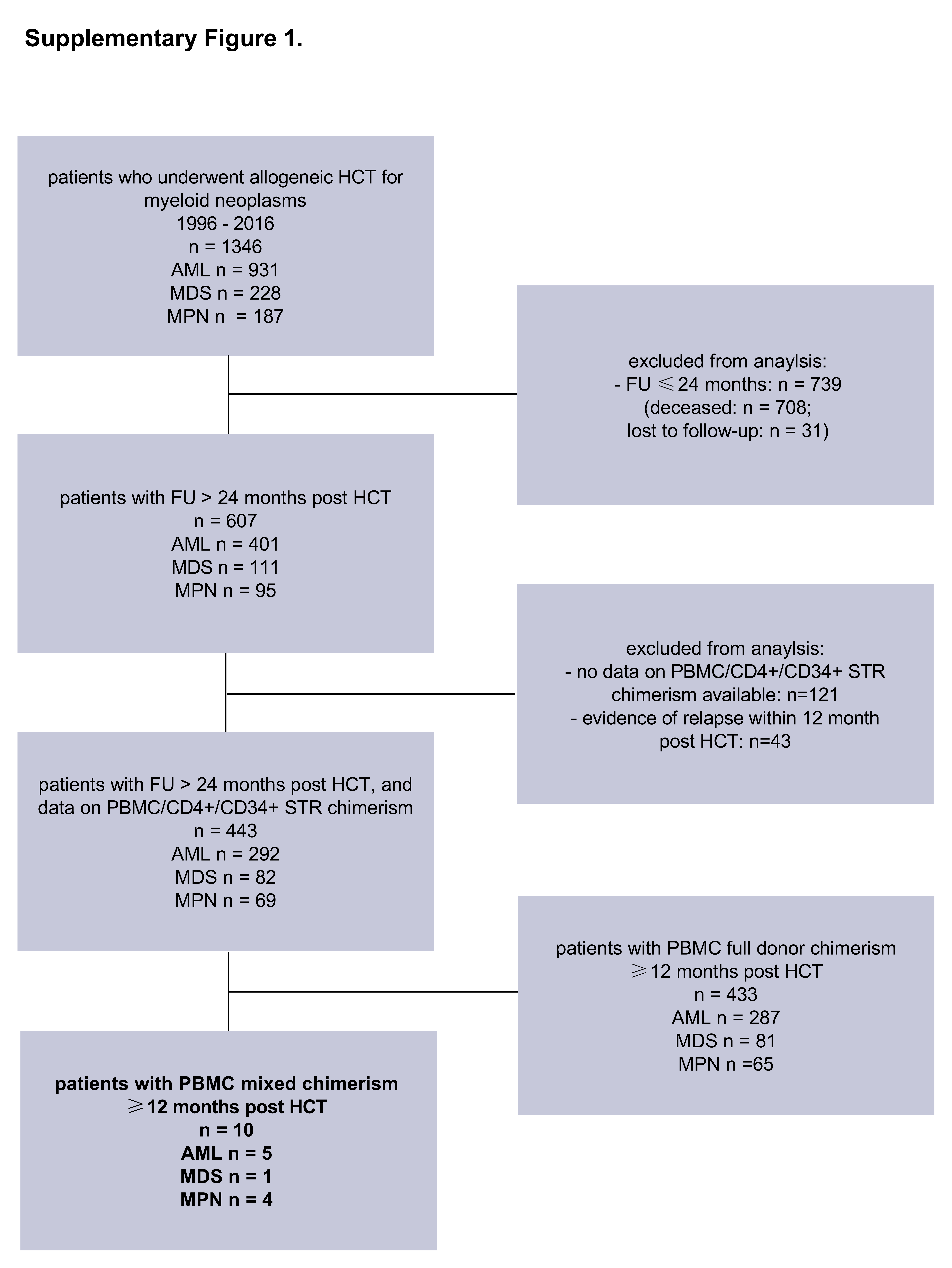

Supplement: Supplementary Figure 1 — Patient selection and composition. AML, acute myeloid leukemia; CML, chronic myeloid leukemia; FU, follow-up; HCT, hematopoietic cell transplantation; MDS, myelodysplastic syndrome; MPN, myeloproliferative neoplasms; PBMC, peripheral blood mononuclear cells; STR, short tandem repeats. [file Image_1.tif]
